# The technical feasibility of an image-guided intensity-modulated radiotherapy (IG-IMRT) to perform a hypofractionated schedule in terms of toxicity and local control for patients with locally advanced or recurrent pancreatic cancer

**DOI:** 10.1186/1748-717X-7-203

**Published:** 2012-12-05

**Authors:** Seok Hyun Son, Jin Ho Song, Byung Ock Choi, Young-nam Kang, Myung Ah Lee, Ki Mun Kang, Hong Seok Jang

**Affiliations:** 1 Department of Radiation Oncology, College of Medicine, The Catholic University of Korea, Seoul, Korea; 2 Department of Internal Medicine, College of Medicine, The Catholic University of Korea, Seoul, Korea; 3 Department of Radiation Oncology, College of Medicine, Gyeongsang National University, Jinju, Korea

**Keywords:** Unresectable pancreatic cancer, Hypofractionated radiotherapy, Image-guided, Intensity-modulated radiotherapy

## Abstract

**Background:**

The purpose of this study was to evaluate the technical feasibility of an image-guided intensity modulated radiotherapy (IG-IMRT) using involved-field technique to perform a hypofractionated schedule for patients with locally advanced or recurrent pancreatic cancer.

**Methods:**

From May 2009 to November 2011, 12 patients with locally advanced or locally recurrent pancreatic cancer received hypofractionated CCRT using TomoTherapy Hi-Art with concurrent and sequential chemotherapy at Seoul St. Mary’s Hospital, the Catholic University of Korea. The total dose delivered was 45 Gy in 15 fractions or 50 Gy in 20 fractions. The target volume did not include the uninvolved regional lymph nodes. Treatment planning and delivery were performed using the IG-IMRT technique. The follow-up duration was a median of 31.1 months (range: 5.7-36.3 months).

**Results:**

Grade 2 or worse acute toxicities developed in 7 patients (58%). Grade 3 or worse gastrointestinal and hematologic toxicity occurred in 0% and 17% of patients, respectively. In the response evaluation, the rates of partial response and stable disease were 58% and 42%, respectively. The rate of local failure was 8% and no regional failure was observed. Distant failure was the main cause of treatment failure. The progression-free survival and overall survival durations were 7.6 and 12.1 months, respectively.

**Conclusion:**

The involved-field technique and IG-IMRT delivered via a hypofractionated schedule are feasible for patients with locally advanced or recurrent pancreatic cancer.

## Background

Pancreatic cancer is the fourth leading cause of cancer death in the United States and the fifth leading cause of cancer death in South Korea [1]. Surgery remains the only potentially curative treatment modality for pancreatic cancer, with a 5-year survival rate of approximately 20% [2]. However, according to the Surveillance, Epidemiology and End Results database, 26% of pancreatic cancer cases are locally advanced at the time of diagnosis, with a 5-year survival rate of 8.7% [3].

The current standard treatment of unresectable locally advanced pancreatic cancer is either concurrent chemoradiation (CCRT) or chemotherapy alone. The clinical results from these treatments showed a poor median survival of 8–14 months [4-16].

Recently, there have been considerable technological advances in the field of radiation oncology such as image-guided, intensity-modulated radiotherapy (IG-IMRT). Due to these advances, more accurate irradiation that delivers a higher dose to the target volume can be achieved with a reduction of the dose delivered to the surrounding normal tissues.

As gemcitabine is known as a potent radiosensitizer, it was combined with radiotherapy (RT) in several studies. However, the maximal tolerated dose of radiation that could be administered with concurrent full-dose gemcitabine was 36 Gy, which was not sufficient for adequate local control and the clinical outcomes were suboptimal [16,17].

We hypothesized that hypofractionated CCRT would improve the local control rate and shorten the treatment duration, which would facilitate the delivery of systemic chemotherapy earlier as compared to conventional RT. Further, IG-IMRT would permit the delivery of a high dose of radiation without an increase in gastrointestinal toxicity. In the present study, we aimed to evaluate the technical feasibility of an IG-IMRT involved-field technique to perform a hypofractionated schedule in patients with locally advanced or recurrent pancreatic cancer.

## Methods

### Patients

Between May 2009 and November 2011, 12 patients with unresectable locally advanced or recurrent pancreatic cancer received hypofractionated CCRT using TomoTherapy Hi-Art at Seoul St. Mary’s Hospital, the Catholic University of Korea. The patients’ data were retrospectively reviewed following institutional review board approval (IRB of Seoul St. Mary's Hospital, The Catholic University of Korea, Reference number: KC11RISI0454). Written informed consent was obtained from the patient for publication of this report and any accompanying images. The inclusion criteria for this study were as follows: 1) a histologically confirmed adenocarcinoma of the pancreas; 2) an unresectable locally advanced disease at the time of diagnosis or locally recurrent disease after a curative resection; 3) patient age >18 years; 4) an Eastern Cooperative Oncology Group performance status of 0, 1, or 2; 5) an adequate bone marrow functional reserve (leukocytes > 3000/μL, an absolute neutrophil count > 1500/μL and a platelet count > 100000/μL); and 6) an adequate hepatic and renal functional reserve (total bilirubin < 1.5 × the institutional limits, aspartate aminotransferase/alanine aminotransferase < 2.5 × the institutional limits and creatinine within the institutional limits). Patients were excluded if they had distant metastases or resectable disease or if they had previously received RT in the upper abdomen.

All patients were evaluated by a multidisciplinary team. The pretreatment evaluation included a medical history, physical examination, complete blood count (CBC), blood chemistry panel, assessment of carbohydrate antigen (CA 19–9) levels, chest X-ray and a pancreas-protocol computed tomography (CT). Magnetic resonance imaging (MRI) and positron emission tomography-computed tomography (PET-CT) were performed in selected patients. Tumors were considered unresectable if they demonstrated any of the following features: 1) a superior mesenteric artery or celiac encasement greater than 180°; 2) an unreconstructable superior mesenteric vein/portal occlusion; or 3) aortic invasion.

### Radiotherapy

For simulation and treatment, patients were immobilized using the BodyFix system (Medical Intelligence, GmbH, Schwabmunchen, Germany), in which the abdomen was compressed with low pressure using foil. A spiral CT scan was then performed with oral and intravenous contrast and a 2.5 mm slice thickness, and 4-dimensional (4D) CT was also performed to assess the movement of the target during respiration using a SOMATOM (Siemens, Berlin, Germany) CT scanner and an ANZAI HW Respiratory Gating System (Siemens, Berlin, Germany).

The gross tumor volume (GTV) was defined as the gross disease identified in the planning CT. For each patient, a 4D CT was performed to determine the adequate margin for the planning target volume (PTV). We measured respiration-induced tumor movement quantitatively using the *syngo* Multi-Modality Workplace (*syngo* InSpace4D, Siemens, Erlangen, Germany) and then created the ITV by adding 7–12 mm to the GTV according to this measurement of the tumor movement. The PTV was generated by adding 3 mm to the ITV with allowing an asymmetric margin expansion in order to reduce irradiation to the stomach, duodenum, and small intestine. The uninvolved regional lymph nodes (LNs), which were traditionally included in conventional RT, were not included in the target volume. This was due to the relatively lower rate of regional failure and our concern about an increased risk of unacceptable toxicities to the surrounding normal tissue, which would have occured if these areas were included in the target volume.

**Figure 1:**
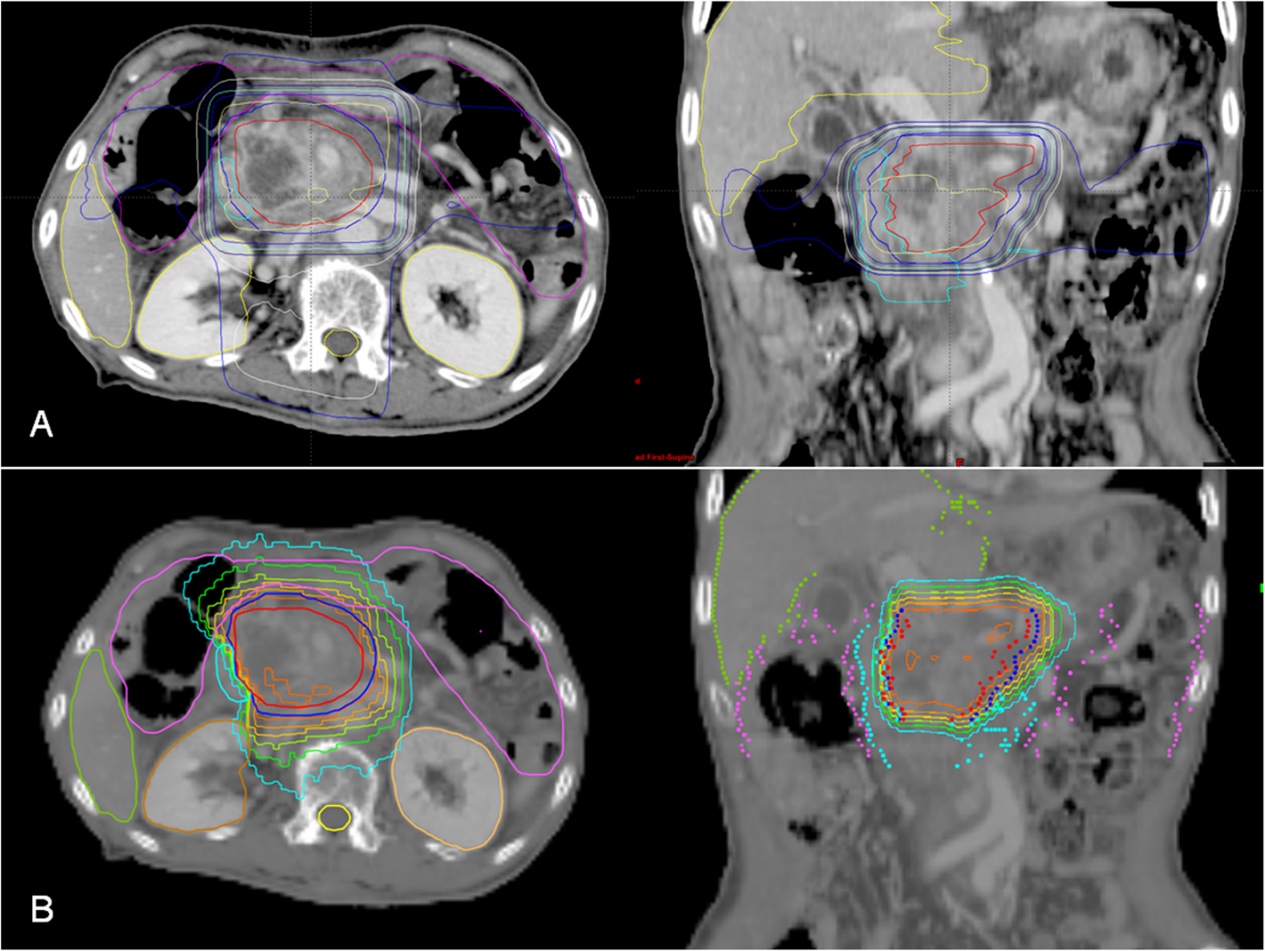
**A comparison of the isodose distribution and dose-volume histogram of intensity modulated radiotherapy (IMRT) and the 4-field technique.** The isodose lines reading from lowest to highest are 50%, 60%, 70%, 80%, 90% and 100% for the 4-field technique (**A**) and IMRT planning (**B**).

We prescribed 45 in 15 fractions or 50 Gy in 20 fractions to 95% of the PTV. Because of concerns of the acute or late gastrointestinal toxicities, we prescribed 45 Gy in 15 fractions to the small PTV and 50 Gy in 20 fractions to the large PTV. The dose constraints of the normal tissue were also used for treatment planning. The volume of liver receiving >25 Gy should be <60% and the mean dose to the liver should be <30 Gy. The volume of each kidney receiving >18 Gy should be <33% and the mean dose to the each kidney should be <16 Gy. The maximal dose to a 2 cc volume (D_2cc_) of the spinal cord, stomach, small intestine, large intestine, and heart should be <42, 45, 45, 48, and 45 Gy, respectively. Treatment planning was performed using the built-in software of the TomoTherapy Planning Station, which was used for the TomoTherapy Hi-Art (Figure 1). We evaluated the dose volume histograms (DVHs) and the dose distributions slice by slice. We then approved the treatment plan if the tumor coverage was adequate and the doses to the surrounding normal tissue were within the proper levels.

Radiation was delivered using a TomoTherapy Hi-Art. Prior to the actual beam delivery, megavoltage cone-beam CT was performed at the time of every treatment session. The patients’ set-up and position were corrected with automated image registration, and the anatomical accuracy was always evaluated by a radiation oncologist.

### Chemotherapy

Chemotherapy was administered concurrently with RT and then sequentially. For CCRT, 12 patients were treated with 5-fluorouracil (5-FU) (600 mg/m^2^/week, weekly). After completing CCRT, 10 patients were treated with gemcitabine (1000 mg/m^2^/week, 3 weeks on and 1 week off) until disease progression and one patient was treated with 5-FU/leucovorin (5-FU: 425 mg/m^2^/day, LV: 20 mg/m^2^/day, days 1–5, monthly). One patient was not treated with chemotherapy after CCRT due to a poor performance status. An average of 4.5 cycles (range: 3–6 cycles) of chemotherapy were administered prior to tumor progression.

### Response and follow-up evaluation

All patients were examined during CCRT and every 2–4 months after the completion of CCRT by a radiation oncologist and a medical oncologist. A physical examination, CBC, blood chemistry panel, assessment of CA 19–9 levels and pancreas-protocol CT were performed at every visit. MRI, PET-CT and esophagogastroduodenoscopy (EGD) were optionally performed.

Tumor response and disease progression were evaluated using the Response Evaluation Criteria In Solid Tumors version 1.1 and the toxicities were evaluated using the Common Terminology Criteria for Adverse Events version 3.1. The co-primary end-points were the toxicity and local response. The secondary end-points were the time to progression (TTP), progression-free survival (PFS) and overall survival (OS).

### Statistical analysis

Statistical analysis was conducted using SPSS version 12.0 software (SPSS Inc., Chicago, IL) and a *p* value <0.05 was considered significant. The TTP, PFS, and OS were estimated using the Kaplan-Meier method. The data was analyzed in May 2012.

## Results

### Patient and treatment characteristics

The patients and their treatment characteristics are summarized in Table 1. All patients included in this study met the inclusion criteria. Of the 12 patients, 9 patients had unresectable locally advanced pancreatic cancer and 3 patients had locally recurrent disease after curative resection (distal pancreatectomy was performed in 2 patients and Whipple’s operation was performed in 1 patient). The PTV were 143.3 ± 5.3 cm^3^ in the patients treated with 45 Gy in 15 fractions and 203.3 ± 57.4 cm^3^ in the patients treated with 50 Gy in 20 fractions. This difference was statistically significant (*p* = 0.014). Regarding the DVHs, the median delivered dose to 95% of the GTV was 51.7 Gy (range: 48.0-58.6 Gy), and that to the D_2cc_ of the stomach, duodenum and small intestine was 44.5 Gy (range: 29.0-52.4 Gy). Although the D_2cc_ of the stomach, duodenum and small intestine was higher than the constraint levels in 5 patients, the treatment plans were accepted to achieve adequate tumor coverage.

**Table 1:** Patient and treatment characteristics

**No.**	**Sex**	**Age**	**Site**	**Stage**	**RT**	**Chemotherapy**		**Local response**	**Site of failure**	**TTP (months)**	**Survival (months)**
					**Dose (Gy/Fxs)**	**CCRT**	**After CCRT**				
1	M	42	Head	T4N1	50/20	5-FU	GEM	SD	Distant	7.6	12.1^*^
2	M	64	Head	T4N0	50/20	5-FU	GEM	PR			7.6^*^
3	M	72	RM	Recurrent^†^	50/20	5-FU	GEM	SD	Distant	7.9	26.0^*^
4	F	61	Head	T4N0	50/20	5-FU	GEM	PR			7.2^*^
5	M	74	Body	T4N0	50/20	5-FU	GEM	SD	Distant	2.0	9.8^*^
6	M	63	Tail	T4N0	50/20	5-FU	GEM	PR	Distant	6.0	9.2^*^
7	F	61	Celiac trunk	Recurrent^†^	50/20	5-FU	5-FU/LV	PR	Distant	8.0	12.8^*^
8	M	66	Body	T4N1	50/20	5-FU		SD	Local/Distant	14.6	22.7^*^
9	M	71	Body	T4N0	50/20	5-FU	GEM	PR	Distant	13.9	19.4^*^
10	M	68	Head	T4N0	45/15	5-FU	GEM	PR	Distant	8.0	20.0^*^
11	F	56	Celiac trunk	Recurrent^†^	45/15	5-FU	GEM	PR	Distant	4.9	6.9
12	F	66	Head	T4N1	45/15	5-FU	GEM	SD			5.7

Abbreviations: RT = radiotherapy; CCRT = concurrent chemoradiation; GEM = gemcitabine; 5-FU = 5-fluorouracil; LV = leucovorin; TTP = time to progression; PR = partial regression; SD = stable disease; RM = resection margin.

^*^ Dead.

^†^ Recurrent disease after curative resection.

### Toxicities

Grade 2 acute toxicities developed in 5 patients (42%) and grade 3 acute toxicities developed in 2 patients (17%). Of these, 5 patients (42%) experienced grade 2 or 3 hematologic toxicities (neutropenia or thrombocytopenia) and 2 patients (12%) experienced grade 2 gastrointestinal toxicities (diarrhea or nausea/vomiting). In 4 patients who experienced symptoms such as gastritis after CCRT, EGD was performed. Of those patients, grade 2 gastric ulcer developed in 2 patients (12%). The toxicities are summarized in Table 2. The PTV was correlated marginally with late gastrointestinal toxicities, but the relationship was not statistically significant (*p* = 0.069). There was a trend toward a correlation between acute or late gastrointestinal toxicities and D_2cc_ of the stomach, duodenum, and small intestine, but it was not statistically significant (*p* = 0.08).

**Table 2:** Grade 2 or worse acute and late toxicities

	**Number of patients (%)**		
	**Grade 2**	**Grade 3**	**Total**
Acute toxicities			
Neutropenia	2 (17%)	2 (17%)	4 (33%)
Thrombocytopenia	1 (8%)	1 (8%)	2 (17%)
Nausea/Vomiting	1 (8%)		1 (8%)
Diarrhea	1 (8%)		1 (8%)
Late toxicities			
Gastric ulcer	2 (17%)		2 (17%)

### Local response and failure-pattern

At the initial response evaluation after CCRT, partial response (PR) and stable disease (SD) were achieved in 7 and 5 patients (58% and 42%), respectively, and none of the patients developed progressive disease. These response evaluations were performed using follow-up CT. In addition, 7 patients (58%) experienced abdominal pain at the time of diagnosis and needed to take analgesics for pain-control, and all of them exhibited improvement of their symptoms after CCRT.

After a median follow-up of 31.1 months (range: 5.7-36.3 months), 1 patient (8%) experienced local progression, and 9 patients (75%) experienced distant progression. No patient experienced regional failure. The median TTP was 8.0 months (95% CI: 7.7-8.3 months). The sites of distant failure were the liver for 4 patients, lung for 4 patients, peritoneum for 5 patients, and bone for 1 patient. The median PFS and OS were 7.6 (95% CI: 6.8-8.3 months) and 12.1 months (95% CI: 7.4-16.7 months), respectively. However, the PFS and OS from this study could not be compared with those of previous reported studies and a significant clinical impact could not be revealed due to the small number of patients included in this study.

## Discussion

The purpose of this study was to evaluate the technical feasibility of an IG-IMRT involved-field technique to perform a hypofractionated schedule in patients with locally advanced or recurrent pancreatic cancer. The hypofractionation regimen used in this study was 45 Gy in 15 fractions or 50 Gy in 20 fractions, which were equivalent to 49.6 or 53 Gy in 1.8 Gy fractions, respectively (assuming an α/β ratio of 10). The CCRT was completed in only 3 or 4 weeks, which was shorter than the 6 weeks necessary for conventional RT. In previously reported studies, a 40–60 Gy dose of radiation was used for conventional RT along with the concurrent use of chemotherapy [5-15], and according to the National Cancer Care Network guideline, a dose of 45–54 Gy of RT in conventional fractionation with the concurrent use of chemotherapy is considered to be standard treatment.

Gemcitabine is a chemotherapeutic agent that has been chosen as the first-line drug for the treatment of pancreatic cancer and has the clinical benefit of a modest survival advantage over 5-FU [18]. In addition, gemcitabine is known as a potent radiosensitizer [19,20]. Therefore, gemcitabine was combined with RT in several studies [11-15]. However, the clinical outcomes in these studies did not reveal the advantage of gemcitabine based CCRT compared with 5-FU based CCRT. Wilkowski *et al.* tested 3 different CCRT regimens (5-FU based CCRT vs. gemcitabine/cisplatin based CCRT vs. gemcitabine/cisplatin based CCRT followed by gemcitabine/cisplatin) [21], and concluded that RT in combination with gemcitabine/cisplatin was not more clinically efficacious than RT with 5-FU because the median OS was similar and grade 3/4 toxicities were more frequent in the 2 gemcitabine/cisplatin arms. According to the study by McGinn *et al.*, the maximal tolerated dose of radiation that could be administered with a concurrent full-dose gemcitabine was 36 Gy in 2.4 Gy fractions [17]. However, this radiation dose was not sufficient for adequate local control. Therefore, we treated used 5-FU as the chemotherapeutic agent along with concurrent CCRT, and full-dose gemcitabine was used sequentially after CCRT.

**Figure 2:**
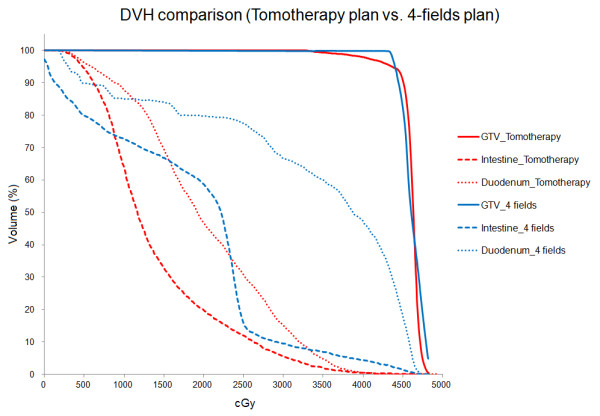
**Dose-volume histograms for GTV, duodenum, and intestine from 4-field technique and IMRT are shown.** Although the doses delivered to the GTV for each treatment plan were similar, there was a large difference between the doses delivered to duodenum and intestine for the treatment plans.

In this study, we excluded the uninvolved regional LNs from the target volume and all patients were treated with IG-IMRT. Murphy *et al.* conducted a study involving highly conformal treatment fields that included only the primary tumor plus a 1 cm margin without the regional LNs, and they reported that only 5% of the patients indicated failure in the peripancreatic LNs and that a larger PTV may not have prevented this failure [16]. Our results indicated that none of the patients experienced any regional failure. The traditional 3 or 4-field technique or 3-dimensional conformal RT was commonly used in previous studies, and there were considerable toxicities from high dose RT when employing these methods [15,22]. The difference between IMRT and traditional 4-field techniques is shown in Figure 2. Although the doses delivered to the GTV for each treatment plan were similar, there were a large difference between the doses delivered to the duodenum and intestine. These differences could explain our lower incidence of gastrointestinal toxicities compared with previous studies using 3–4 fields or conformal techniques. In a recent Federation Francophone de Cancerologie Digestive/French Society of Radiation Oncology (FFCD/SFRO) study, the target volume included the primary tumor plus the uninvolved regional LNs with a wide margin and the conformal RT technique was used with high dose radiation (60 Gy) [22]. The FFCD/SFRO study did not identify an advantage for RT on an interim analysis because of the high incidence of toxicity. Therefore, excluding uninvolved regional LNs from the target volume for hypofractionated CCRT is deemed a proper strategy when it is combined with effective chemotherapy. IG-IMRT could provide adequate target coverage and reduce the incidence of serious toxicities.

The stomach, duodenum, and small intestine adjacent to a pancreatic mass are dose-limiting organs. If the dose delivered to these organs was not carefully planned according to the constraint levels or if the dose was inaccurately delivered, then the risk of gastrointestinal toxicities such as ulcer, bleeding, stricture, obstruction and perforation could be increased. The incidence and severity of gastrointestinal toxicities in this study was thought to be acceptable compared with those in previous studies. In this study, grade 3 or worse gastrointestinal and hematologic toxicity were 0% and 17%, respectively. These rates were similar to or slightly lower than the findings of previous studies using 5-FU based CCRT [7-10] and significantly lower than the findings of previous studies using gemcitabine based CCRT [12-16]. This is summarized in Table 3. In addition, 2 patients (17%) experienced medically controllable grade 2 gastric ulcers. None of the patients experienced any grade 3 or worse late toxicities. This is lower than 2-11% of grade 3 or worse late gastrointestinal toxicities in patients treated with gemcitabine based CCRT with conventional or hypofractionation regimen [14-16].

**Table 3:** Results of concurrent chemoradiation

**Author**	**Treatment regimen**	**No. of patients**	**Gastrointestinal toxicity (≥ Grade 3)**	**Hematologic toxicity (≥ Grade 3)**	**Local failure rate**	**Median PFS (months)**	**Median OS (months)**
Cohen *et al.*[7]	RT 59.4 Gy/5-FU+mitomycin	55	13%	20%	NA	5.1	8.4
Ishii *et al.*[8]	RT 50.4 Gy/5-FU	20	15%	0%	35%	4.9	10.3
Kornek *et al.*[9]	RT 55 Gy/5-FU+cisplatin	38	18%	18%	NA	10	14
Boz *et al.*[10]	RT 59.4 Gy/5-FU	42	4%	16%	51%	6.2	9.1
Magnino *et al.*[12]	RT 45 Gy/GEM	23	30%	39%	22%	NA	12
Blackstock *et al.*[13]	RT 50.4 Gy/GEM	39	41%	69%	15%	NA	7.9
Okusaka *et al.*[14]	RT 50.4 Gy/GEM	38	33%	52%	6%	4.4	9.5
Li *et al.*[15]	RT 50.4-61.2 Gy/GEM	18	28%	28%	34%	7.1	14.5
Murphy *et al.*[16]	^*^RT 36 Gy/GEM	74	11%	24%	26%	NA	11.2
This study	^*^RT 50 Gy/5-FU	10	0%	17%	8%	7.6	12.1

Abbreviations: PFS = progression-free survival; OS = overall survival; RT = radiotherapy; 5-FU = 5-fluorouracil; GEM = gemcitabine; NA = not available.

^*^ Hypofractionated CCRT.

At the response evaluation, 7 patients (58%) achieved a PR, and 5 patients (42%) exhibited a SD. The treatment response was determined by the reduced size of the mass in the follow-up CT. However, the response for a treated pancreatic mass can be underestimated because the size reduction on the anatomic imaging cannot be perfectly assessed due to varying degrees of acute or subacute pancreatitis or radiation-induced fibrosis. Local progression of a primary mass may cause severe abdominal pain, gastric outlet/duodenal obstruction, and bleeding which adversely influence the quality of life and can be a cause of death in many cases; therefore, control of the primary tumor is extremely important. In this study, 1 patient (8%) experienced local progression. The major cause of treatment failure was distant metastases, which developed in 10 patients (83%). In addition, 6 of our patients who experienced abdominal pain at the time of diagnosis indicated improvement of their symptoms after CCRT. Therefore, this hypofractionation regimen can be considered an effective treatment for pain control and local control compared with the local failure rate of 6-51% reported for conventional RT [3,8,10,12-15]. Because of the small number of patents included in this study, the PFS and OS from this study could not be compared with those of previously reported studies, and therefore, the clinical significance of our findings could not be demonstrated (Table 3).

In conclusion, the involved field technique and IG-IMRT using TomoTherapy provided adequate target coverage while sparing the surrounding normal tissues and consequently reduced the incidence of serious gastrointestinal toxicities and the rate of locoregional failures. Therefore, it is feasible to perform a hypofractionated schedule in patients with locally advanced or recurrent pancreatic cancer.

## Competing interests

The authors declare that they have no competing interests.

## Authors’ contribution

SHS, JHS, BOC and HSJ performed all CT evaluations, target contouring, data collection and interpretation of the data. YNK performed the treatment planning and conducted all planning evaluations. SHS, JHS, HSJ and MAL took care of the patients. SHS, MAL, KMK and HSJ participated in the study design. SHS performed the statistical analysis and drafted the manuscript. All authors read and approved the final draft.
